# Calcitonin Gene-Related Peptide (Cgrp), Adrenomedullin (Am), Amylin, And Calcitonin (Ct) Receptors And Overlapping Biological Actions

**DOI:** 10.1100/tsw.2001.413

**Published:** 2001-10-26

**Authors:** J.A. Fischer, W. Born, R. Muff

**Affiliations:** Research Laboratory for Calcium Metabolism, University of Zurich, Klinik Balgrist, 8008 Zurich, Switzerland

## Abstract

CGRP, AM, amylin, and CT have in common N-terminal 6-7 amino acid ring structures linked by disulfide bridges and amidated C-termini required for biological activity. For the related bioactive peptides, receptor-binding sites linked to cAMP stimulation and to a lesser extent to the phospholipase C signaling pathway have been identified in tissue specific manner. The highest density of CGRP receptors has been recognized in the cerebellum and the spinal cord. There photoaffinity-labeled N-glycosylated 60,000 and 54,000 Mr proteins are converted to 46,000 and 41,000 Mr components following endoglycosidase F/N-glycosidase F treatment. The same proteins were specifically labeled with [125I]-hCGRP-I(1-37) and -(8-37). Some cross-reaction between the CGRP receptor and AM was evident whereas amylin and CT were only recognized at over 10-7 M. A different AM receptor localized predominantly in the lung recognized CGRP at low, and amylin and calcitonin at equally high concentrations. CT receptor binding sites have been identified in osteoclasts and in the periventricular region of the brain. They cross-reacted with amylin at low concentrations and with CGRP and AM at over 10-7 M. Amylin receptor binding sites cross-reacting with salmon CT and CGRP but not with hCT and adrenomedullin to any great extent were originally described by Sexton in the nucleus accumbens and may represent a second CGRP receptor. The structure of a CT receptor was elucidated by the group of Goldring in 1991 through molecular cloning, and of a 60% homologous human CT receptor-like receptor (CRLR) shortly thereafter here. The latter was an orphan receptor until the discovery of the receptor-activity-modifying proteins (RAMP) by Foord which upon coexpression yield a CGRP receptor with RAMP1 and an AM receptor with RAMP2. Coexpression of the hCT receptor isotype 2 revealed a CGRP/amylin receptor with RAMP1 and an amylin receptor isotype with RAMP3. The CRLR/RAMP1 receptor antagonized by CGRP(8-37) corresponds to the CGRP1 receptor defined by Quirion, whereas his CGRP2 receptor remains to be identified. Another CGRP receptor isotype remains to be discovered in the cerebellum with no detectable CRLR encoding mRNA. Overlapping biological actions include inhibition of bone resorption obtained predominantly with CT, but also at high concentrations with CGRP, AM, and amylin. CGRP and AM are potent vasodilators, an effect shared with CT at pharmacological concentrations. Biological actions of amylin include suppression of insulin secretion, stimulation of glycogenolysis and inhibition of glycogen synthesis. In conclusion, the hCT2 receptor or the CRLR are associated with one of three RAMPs to bind to CGRP, AM, or amylin.

